# Zinc Affects Cholesterol Oxidation Products and Fatty Acids Composition in Rats’ Serum

**DOI:** 10.3390/nu13051563

**Published:** 2021-05-06

**Authors:** Agnieszka Stawarska, Małgorzata Czerwonka, Rafał Wyrębiak, Robert Wrzesień, Barbara Bobrowska-Korczak

**Affiliations:** 1 Department of Bromatology, Faculty of Pharmacy, Medical University of Warsaw, Banacha 1, 02-097 Warsaw, Poland; agnieszka.stawarska@wum.edu.pl (A.S.); malgorzata.czerwonka@wum.edu.pl (M.C.); 2 Department of Biomaterials Chemistry, Chair of Analytical Chemistry and Biomaterials, Faculty of Pharmacy, Medical University of Warsaw, Banacha 1, 02-097 Warsaw, Poland; rafal.wyrebiak@gmail.com; 3 Central Laboratory of Experimental Animals, Medical University of Warsaw, Poland, Banacha 1a, 02-091 Warsaw, Poland; robert.wrzesien@wum.edu.pl

**Keywords:** zinc, fatty acids, Δ6- and Δ5–desaturase, cholesterol, oxysterols

## Abstract

The purpose of this work was to evaluate the effect of the nanosized or microsized zinc (Zn) particles on fatty acid profile, enzyme activity and the level of cholesterol, squalene and oxysterols in rats with breast cancer. Rats (female, *n* = 24) were divided into the following groups: control, and two test groups, whose diets were enriched with either Zn microparticles (342 nm) or Zn nanoparticles (99 nm). All rats were treated twice with the carcinogenic agent; 7,12-dimethylbenz[a]anthracene. In rats whose diet was enriched with zinc (especially in the form of nanoparticles), the number and sizes of tumors were lower. Diet supplementation also significantly reduced the cholesterol (*p* = 0.027) and COPs (cholesterol oxidation products) levels (*p* = 0.011) in rats serum. Enriching the diet with Zn microparticles decreased the Δ6-desaturase activity (*p* < 0.001). Zn influences fatty acids’ profile in rats’ serum as well as inhibiting desaturating enzymes. A reduced amount of pro-inflammatory arachidonic acid derivatives may be the expected effect.

## 1. Introduction

The fatty acids profile in the body depends on fatty acids intake with a diet, their endogenous synthesis and the activity of enzymes responsible for the metabolism of fatty acids. These processes are also influenced by both physiological and pathological conditions that occur in the body. The increase of linoleic acid’s (LA) supply in the diet causes the increase of the amount of arachidonic acid (AA), which is known to be the main substrate of pro-inflammatory eicosanoids (e.g., prostaglandin E2). At the same time, the transformation of acids of the n-3 series weakens. Analogically, when the α-linolenic acid (ALA) consumption increases, the increase in the quantity of n-3 acids follows, while the synthesis of n-6 acids decreases. This is due to the competition between families for the same enzymes. Polyunsaturated fatty acids have an important effect in many pathological conditions. Studies show that some fatty acids, e.g., LA, can stimulate the carcinogenic process, but others, e.g., γ-linolenic acid (GLA), eicosapentaenoic acid (EPA), and docosahexaenoic acid (DHA) can inhibit this process. Desaturases activity plays a crucial part in both LA and ALA metabolism. 

So far it has been shown that different factors related to the diet can impact the activity of these enzymes [1,2,3,4]. Based on the current state of knowledge, it can be assumed that the activity of enzymes can also be influenced by selected minerals present in the diet [5]. Studies show a relationship between Δ5-desaturase (D5D) and Δ6-desaturase (D6D) activity and the occurrence of several diseases such as cancer, diabetes, obesity and other inflammatory diseases [6,7,8]. However, the results of these studies are inconclusive. Therefore, further research is necessary.

Zinc is a micronutrient for numerous metabolic processes. It is used in about 300 biochemical and enzymatic processes in the body [9,10,11]. Zinc is part of enzymes such as desaturases and elongases [12,13]. Many studies confirmed the relationship between zinc and the metabolism of fatty acids (FA) [5,14]. Ambiguous data show the influence of zinc on the human organism, especially in pathological conditions. The use of zinc in nanomedicine is of great interest. Nanoparticles can be helpful in the treatment of many diseases [15,16,17,18,19]. There are reports of potentially beneficial effects of zinc, particularly in the form of nanoparticles in cancer treatment [15,18,19], unfortunately, there is still a lack of in vivo studies. All the more interesting is the influence of this element, depending on the form in which it is administered, in the prevention and development of the cancer process. Moreover, new mechanisms of its action are still being sought. There is a lack of data on the effect of zinc in the form of nanoparticles on fatty acids composition and metabolism.

Oxysterols are derivatives of cholesterol. There are two main sources of them in the human body. The first is endogenous synthesis in vivo in cells, through cholesterol metabolism. The second exogenous source is the food we eat. Endogenous oxysterols can be formed by non-enzymatic (free radicals) or enzymatic mechanism involving cholesterol hydroxylases of the cytochrome P-450 family [20]. In the body, oxysterols contribute to the intensification of inflammatory processes both by inducing the expression as well as enhancing the synthesis of pro-inflammatory cytokines [tumor necrosis factor (TNF-α), interleukin 1β (IL-1β) and 6 (IL-6)], chemokines [monocyte chemotactic protein-1 (MCP-1)], adhesion particles and activation of phagocytes. Cholesterol oxidation products (COPs) are one of the factors influencing the activation of cytokine-dependent inflammatory processes [20,21,22]. Inflammation is a contributing cause to the development of many disease entities. Oxysterols have been suggested to play a role in a variety of clinical settings, including diabetes mellitus type 2, atherosclerosis, neurodegenerative disorders (Alzheimer’s disease, Parkinson’s disease, Huntington’s disease) and cancer (such as breast, prostate, colon, and bile duct) [20,21,22]. It seems promising to use the level of a given oxysterol in the body to assess the risk of a given disease.

The project aimed to determine the effect of zinc dietary supplementation in the form of nano- or microparticles on the fatty acids composition and activity of desaturases (D6D, D5D) in rats under the conditions of the cancer process. The level of cholesterol and oxysterols was also determined.

## 2. Materials and Methods

### 2.1. Micro- and Nanoparticles of Zinc

The manufacture of Zn particles was described in our previous publication [23].

### 2.2. Animals

The experiment was conducted on 24 Spraque-Dawley female rats. During the treatment, all animals had unlimited access to fodder and water (Labofeed H fodder, Table 1). The average daily energy consumption was approximately 30 kcal (average of 12 g of fodder per day). Animals were kept in a room with constant air humidity and temperature of 23 °C. The 12 h cycle of light and dark was respected.

After a 1-week adaptation period, the rats (40 days old) were divided into three groups (8 animals in each group) depending on the type of diet:

- CON—control group,
- ZnM—group receiving zinc in the form of microparticles (342 nm),
- ZnN—group receiving zinc in the form of nanoparticles (99 nm).

Both micro- and nanoparticles of zinc were administered *via gavage* in the dose of 4.6 mg/mL in 0.4 mL of water, started on the 40th day of life for 20 weeks. To ensure a similar stress level in all tested animals, the control group received 0.4 mL of water *via gavage*. Animals were gavage daily except Saturday and Sunday.

On the 60th and 90th day of life, all rats were treated with a carcinogenic agent—7,12-dimethylbenz[a]anthracene (DMBA), in the amount of 80 mg/kg body weight (b.w.) and 40 mg b.w., respectively. DMBA was given to introduce breast cancer. The tumor-inducing agent was dissolved in rapeseed oil. Starting from the 7th week of life, animals were weighed weekly. Everyday rats were palpated to detect the appearance of tumors and monitored for any specific signs of welfare disorders (e.g., appetite loss, sluggishness, hiding).

The rats were decapitated in week 21 of the experiment. The weights of internal organs were determined. The serum was collected from fresh blood and was stored at the temperature of −80 °C until the analysis. 

The study was approved by the relevant animals’ committee (decision no 645/2018).

### 2.3. Preparation of Serum

Serum was acquired by centrifugation of fresh blood at 3000 rpm for 10 min. The collected animal material was stored at the temperature of −80 °C until the analysis.

### 2.4. Determination of Fatty Acids Methyl Esters in Serum

After the methylation procedure, gas chromatography mass spectrometry (GC/MS) was used to determine the concentration and profile of the fatty acid. To 200 μL of serum 1 mL of 0.5 M NaOH in methanol and 25 μL of internal standard (C19:0 in toluene, 1 mg/mL) were added and heated in 80 °C for 15 min. Then 1 mL of BF3 solution in methanol (14% *w*/*v*) was added and again heated in 80 °C for 15 min. Then, extraction of FAME (Fatty Acids Methyl Esters) was performed by adding 2 mL of a saturated solution of NaCl in water and 1 mL of hexane and shaking. After phase separation, the hexane layer was moved to a 2 mL vial, then injected into the column.

Analyses were performed on a gas chromatograph with time-of-flight mass spectrometry (Pegasus® BT, LECO Corporation, St. Joseph, MI, USA). Chromatographic separations were conducted on a capillary column TR-FAME (120 m/0.25 mm ID/film thickness 0.25 μm; Thermo Fisher Scientific Inc., Göteborg, Sweden). Helium was used as the carrier gas (flow: linear velocity at 1 mL/min), the injection was 1 μL, in the splitless mode. The injector and transfer line were heated to 240 °C. The temperature program was as follows: initial temperature 110 °C for 16 min; increase by 6 °C/min to 190 °C; increase 1 °C/min to 210 °C; increase 0.4 °C/min to 220 °C and hold for 10 min in 220 °C. Ion source temperature was 250 °C and energy 70 eV. To recognize the FA contained in samples, the FAME standards (Supelco 37Component FAME Mix, Sigma, St. Louis, MO, USA) were applied.

Knowing the FA compositions in serum, the peroxidability index (PI) of experimental groups was determined [24].

### 2.5. Estimation of Desaturases Activity

The indices of desaturases (D6D and D5D) activity were calculated as the ratio of product to the precursor of FA.

The ratio of γ-linolenic acid concentration (GLA, C18:3, n-6) to the linoleic acid (LA, C18:2, n-6) concentration in serum was the D6D index value, while the ratio of arachidonic acid concentration (AA, C20:4, n-6) to the dihomo-γ-linolenic acid concentration (DGLA, C20:3, n-6) was the index value of D5D.

### 2.6. Determination of Squalene, Total Cholesterol and Oxysterols Content

Squalene, cholesterol, and cholesterol oxidation products (COPs) were determined by gas chromatography with mass spectrometry. Sample preparation consisted of two steps: hydrolysis and silylation. To 200 mL of serum, 3 mL KOH in ethanol (1 M), 25 μL of internal standard solution (5α-cholestane in hexane, 0.5 mg/mL) and 10 μL BHT solution (butylated hydroxytoluene in ethanol, 5 mg/mL) were added. The sample was shaken and left for 20 h. After incubation 4 mL of water, 2 mL of hexane were added and shaken. The hexane layer was collected to a 2 mL vial and evaporated under a stream of nitrogen. The dry residue was dissolved in 50 μL of pyridine and 25 μL silylation mixture (N,O-bis(trimethylsilyl)trifluoroacetamide with 1% of trimethylchlorosilane, for GC derivatization) were added and mixed thoroughly. The sample was incubated at a temperature of 80 °C for 40 min. After cooling to room temperature, 225 μL of hexane were added and the sample was injected into the column. Gas chromatography with time-of-flight mass spectrometry (Pegasus® BT, LECO Corporation, St. Joseph, MI, USA) was used to determine squalene, cholesterol, and its oxidized derivatives. The capillary column Rxi®-17SilMS (30 m × 0.25 mm × 0.25 μm, Restek, Bellefonte, PA, USA) was used. Helium (carrier gas) flow was 1 mL/min. The sample (1 µL) was injected in splitless mode. Injector and transfer line temperature was 290 °C. The temperature program was as follows: initial temperature—200 °C for 4.60 min; increase 5 °C/min to 290 °C; hold 290 °C 12.4 min. Ion source temperature was 250 °C and energy 70 eV. Squalene and trimethylsilyl derivatives of cholesterol and COPs were identified based on retention times and mass spectra.

### 2.7. Statistical Analysis

Data are presented as mean value ± standard deviation. Differences between groups were analyzed using one-way analysis of variance (ANOVA, α = 0.05) with post-hoc Tukey’s test (α = 0.05).

Cluster analysis has also been carried out; the results are presented as heatmaps. Statistical analysis was performed using Statistica 13.3 (StatSoft, Cracov, Poland) software.

## 3. Results

### 3.1. Body and Internal Organs Weight in Rats

**Figure 1:**
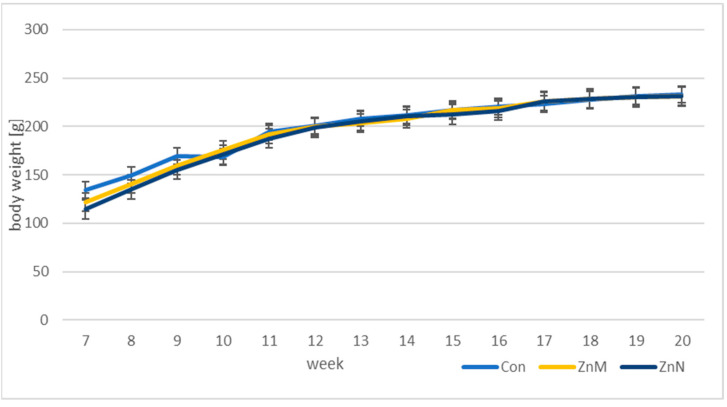
Changes in average body weight of the rats in experimental groups.

The weights of the animals at the beginning of the experiment were slightly different, but at the end of the treatment, no significant differences in this parameter were noted between the groups. The results are shown in Table 2 and Figure 1.

The applied diet modification did not affect the weight of the liver, kidneys, spleen and heart of the examined rats.

### 3.2. The Occurrence of Tumors

Rats whose diets were supplemented with zinc in nanoparticles showed a lower incidence of cancer than the control group. Moreover, in the ZnN group the number of tumors and their masses per animal was lower compared to the control group. Results are presented in Table 3. No tumor metastases to other organs and bones were found in the model used (data based on histopathological examinations).

### 3.3. Fatty Acids Content in Rats’ Serum

**Figure 2:**
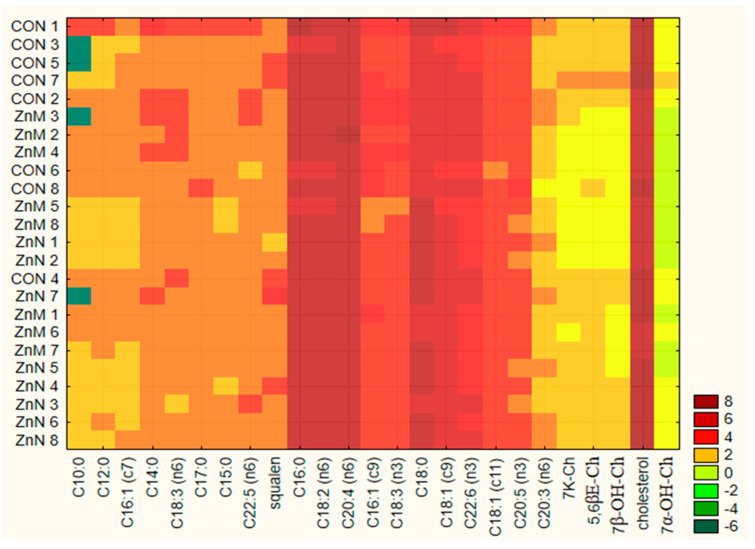
Heatmap for the overview: comparison of fatty acids, squalene, cholesterol and its oxidized derivatives in serum of individual rats.

In the serum of all rats the following acids were predominant: arachidonic (AA, C20:4 n-6), palmitic (C16:0), linoleic (LA, C18:2, n-6), stearic (C18:0) and oleic (OL, C18:1 n-9) (Table 4) (Figure 2). When comparing the content of individual fatty acids in the serum of the studied animals, it was observed that arachidonic acid had the highest share of total fatty acids content. When it comes to the AA amount, no statistically significant differences were observed among the study groups.

The highest contents of palmitic acid (677.3 ± 145.6 μg/mL), stearic acid (510.22 ± 76.5 μg/mL), oleic acid (243.5 ± 32.5 μg/mL), linoleic acid (632.4 ± 72.5 μg/mL) and the lowest level of arachidonic acid (820.9 ± 125.7 μg/mL) were found in the ZnM group.

Among all fatty acids, SFA’s content ranged from 33% to 38% (Table 5), while on average, the lowest content of all saturated acids was found in the ZnN group and the highest in the ZnM group (Table 4). We discovered that the introduction of Zn in the diet resulted in lowering of the share of capric acid (C10:0), lauric acid (C12:0), myristic acid (C14:0) and hepatadecanoic acid (C17:0) in comparison to CON group. Moreover, supplementing the diet with Zn significantly contributed to the reduction of the percentage share of pentadecaenoic acid (C15:0) in group ZnM and ZnN, compared to the CON group. The highest levels of palmitic and stearic acid were determined in the ZnM group, respectively: 677.3 ± 145.6 μg/mL and 510.22 ± 76.5 μg/mL.

Supplementing the diet with Zn caused the content of monounsaturated (MUFA) and polyunsaturated fatty acids (PUFA) in the serum to decrease, while in the case of saturated acids, different types of supplementation resulted in different changes in their amount. MUFA amounted to about 10% of all fatty acids, and no differences between their total content were observed between groups. Among MUFA, the only acid characterized by its higher content was the oleic acid (C18:1, n-9), but no significant differences in concentration were observed between all groups (Table 4). Supplementing the diet with Zn resulted in the reduction of the content of palmitoleic acid (C16:1, n-7) compared to the CON group (Table 4).

PUFA amounted to 52% to 57% of all fatty acids. The group supplemented with micro Zn was found out to have the lowest total amount of PUFA, which was significantly lower than its content in any of the other groups. Supplementing the diet with micro- and nano-Zn decreased the share of n-3 PUFA significantly, as the concentrations of ALA (C18:3, n-3), EPA (C20:5, n-3) and DHA (C22:6, n-3) in groups ZnN and ZnM were smaller in comparison to CON group (Table 4). In all study groups, regardless of the supplementation used, a similar level of n-6 family acids was observed. A statistically significant increase in the n-6/n-3 ratio, compared to the control group, was observed in groups ZnM and ZnN. Although, the increase in the case of the ZnM group was slightly higher. Moreover, the MUFA + PUFA/SFA and PUFA/SFA ratios were determined. We observed that diet supplementation with Zn microparticles significantly decreased both ratios as well as the peroxidability index (PI).

### 3.4. Desaturases (D6D and D5D) Activity Indices

**Figure 3:**
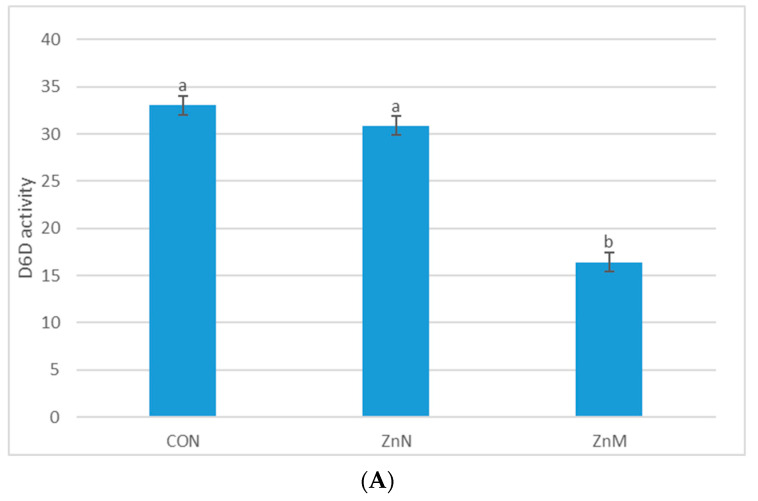

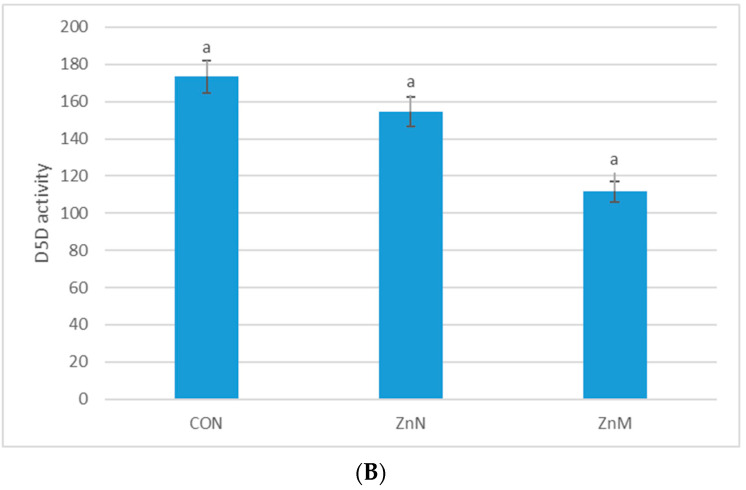
Activities of D6D (**A**) and D5D (**B**) in the serum of experimental groups. CON—control group; ZnN—group receiving zinc in nanoparticles; ZnM—group receiving zinc in microparticles. ^a,b^—homogenous groups (α = 0.05), D6D–Δ6-desaturase index, D5D–Δ5-desaturase index.

The D6D index was calculated as the GLA/LA concentration ratio and the D5D index as the AA/DGLA concentration ratio. The D6D index, showing the enzyme activity, tended to be the highest in the serum obtained from the control group (33.06 ± 8.22) and slightly lower (30.87 ± 9.64) in the animals supplemented with Zn nanoparticles (Figure 3A). Results for the group supplemented with Zn in microparticles show the lowest D6D activity (16.43 ± 4.55). Supplementing the diet with Zn caused a decrease in D5D activity in the serum (Figure 3B). In rats supplemented with Zn, the D5D activity’s value was slightly lower in the ZnN group, but the lowest was observed in the ZnM group. However, these differences are not statistically significant.

### 3.5. Determination of Cholesterol and Oxysterols

The level of cholesterol in all groups was between 979 ± 115 μg/mL (ZnN group) and 2006 ± 1091 μg/mL (CON group) (Table 6). Among these groups, some significant differences in total COPs were observed (Table 6, Figure 1). The total COPs was the highest for the control group. Zinc supplementation significantly decreased the content of COPs, both in the nano- and microparticles groups, 8.15 ± 1.51 μg/mL and 11.95 ± 4.37 μg/mL, respectively. 7-ketocholesterol (7K-Ch), 7β-hydroxycholesterol (7β-OH-Ch) and 5β,6β-epoxycholesterol (5β,6βE-Ch) had the highest contributions to the COPs. Supplementing the diet with both micro- and nanozinc led to decrease the content of all detected oxysterols.

## 4. Discussion

Zinc is a micronutrient that modulates inflammation in the body. It has a strong antioxidant effect, mainly as a component of superoxide dismutase, which can inhibit tumor growth. The results obtained in our experiment show that dietary supplementation with zinc under the conditions of the neoplastic process did not adversely affect the health of animals. No loss of appetite, lethargy or other stress behaviors were observed in them. On the other hand, the supplementation used, especially with zinc in nanoparticles, translated into the number and size of tumors found in rats with DMBA-induced breast cancer. There were fewer of them and they were significantly smaller compared to the control group.

Many factors present in the diet and the general state of the organism can influence the profile of fatty acids in the body. Studies have shown that the occurrence of diseases, including cancer, can have a significant impact [8]. A relationship between FA metabolism and Zn supplementation has been observed in several studies [25,26]. On the other hand, no one has investigated whether the influence of the particle size in which the zinc was applied was of importance. For the first time, the influence of zinc supplementation in the form of micro- and nanoparticles on the metabolism and profile of FA was investigated. Additionally, the influence of such administration on the formation of cholesterol and its derivatives, oxysterols was also determined. 

Palmitic and stearic acids are the most common representatives of saturated fatty acids in the human body. Among observations, the most interesting one concerns rats supplemented with zinc in microparticles. Serum palmitic and stearic acids concentration were the highest for the remaining groups. In this group, a higher percentage of total SFA was found. Research shows that the inflammatory response may be influenced by fatty acids through the activation of nuclear factor κB (NF-κB), leading to the increased synthesis of pro-inflammatory cytokines (interleukin 6, tumor necrosis factor α) as well as to biosynthesis of proinflammatory eicosanoids from arachidonic acid (prostaglandin E2) [27].

Despite different supplementations used in this experiment, the changes in sums of monounsaturated and polyunsaturated fatty acids are not statistically significant. Published research confirmed, that FA composition of phospholipids in rat liver, plasma and erythrocytes was influenced by Zn deficiency [12]. Among all n-6 FA, AA and LA were found to have the highest content. Some studies have reported higher levels of linoleic acid, while some have found lower levels of AA and others high or normal levels of arachidonic acid in the phospholipid tissue of animals with zinc deficiency [12]. To our surprise, the introduction of zinc supplementation in rats resulted in a decrease (ZnN group) and an increase (ZnM group) of LA concentration in rats, with a decrease in the content of AA in all supplemented groups. However, this effect was not statistically significant. Diet supplementation with zinc decreases the activity of desaturases, which leads to inhibiting the transformations of polyunsaturated fatty acids to AA. This leads to a decrease in the synthesis of eicosanoids (AA derivatives). One of them, having immunosuppressive properties, is prostaglandin E2, which is pro-inflammatory and has pro-neoplastic activity, too [7]. On the other hand, the most commonly found n-3 FA in the serum of rats was the DHA. Supplementing the diet decreased its content no matter in which form the zinc was administered in. A similar trend was observed for the EPA.

On the basis of the results, we can see the negative correlation between Zn supplementation and enzyme activity. The highest D6D and D5D indices were observed in the group without supplementation. This indicates that a diet rich in Zn, particularly in microsized particles, inhibits the conversion of fatty acids. On the other hand, supplementing the diet with zinc in the form of nanoparticles does not have such a strong effect. The same effect was also confirmed by other authors [3]. This indicates that a diet supplemented with zinc could significantly inhibit the activity of enzymes, which will influence fatty acid conversion and eicosanoid synthesis. The increased level of PGE2 was found in several types of cancer, e.g., in breast cancer [28].

Lin et al. conclude that exposure to ZnO nanoparticles leads to cytotoxicity reflected in oxidative stress, lipid peroxidation, cell membrane damage, and oxidative DNA damage [29]. It was also found that zinc in the nano form induced the production of free radicals causing oxidative damage, induced the inflammatory process and caused cell death [30].

Oxysterols, which are products of the oxidation of cholesterol, have received increasing attention as a diagnostic biomarker of oxidative stress [31]. Dietary modifications (ZnM and ZnN) result in a significantly decreased cholesterol content in rat serum compared to the control group. In humans with a zinc-supplemented diet, a significant decrease in total cholesterol’s concentration in plasma was observed [32]. In contrast to our results, Katya-Katya et al. [33] found an increase in the concentration of cholesterol after the third month of zinc supplementation in rats. 7-Ketocholesterol, 7β-hydroxycholesterol and 5β,6β-epoxycholesterol were found to be the main COPs. Similar results were obtained in other authors’ studies [33]. The control group was found to have the highest total concentration of COPs. Antioxidant potential of zinc can be the reason for lower content of total COPs in groups receiving dietary supplements, by influencing the formation of COPs which are produced under oxidative stress. It is broadly known that some oxysterols (7-K-Ch, 7α-hydroxycholesterol (7α-OH-Ch), 7β-OH-Ch, 5α6α-epoxidecholesterol, 5,6βE-Ch, 25-hydroxycholesterol), have potent pro-inflammatory properties [22,34,35]. Inflammation contributes to the development of many disease entities. A high level of COPs is associated, among other things, with an increased risk of cancer, including breast cancer. In our study we observed a tendency to significantly decrease the content of 7K-Ch, 7α-OH-Ch, 7β-OH-Ch, 5,6βE-Ch, in serum of rats, especially supplemented with nanoparticles of Zn. This confirms that the size of applied particles influences the biological activity of zinc in vivo.

## 5. Conclusions

This is the first report that demonstrates how zinc in micro- and nanoparticles influences the composition and metabolism of fatty acids, especially the activity of desaturase and the level of cholesterol and COPs in the serum of rats with cancer. The decrease in desaturases’ activity was the result of supplementation used. A beneficial effect of dietary supplementation with Zn, especially in nanoparticles form, on the formation of cholesterol, squalene and COPs was found in this work. This indicates that supplementing the diet with zinc can be an anti-inflammatory mechanism. Moreover, the beneficial effect of zinc supplementation in the form of nanoparticles has been confirmed, which may be promising when developing and using these compounds as dietary supplements.

## Figures and Tables

**Table 1:** Labofeed H—composition (per kg of diet).

Composition of Labofeed H diet (per kg).			
Protein (g)		210.0	
Fat (g)		39.2	
Fiber (g)		43.2	
Starch (g)		300.0	
Ash (g)		55.0	
Vitamin A (IU)	15,000	Vitamin B_6_ (mg)	17.0
Lysine (g)	14.5	Histidine (g)	6.0
Vitamin D_3_ (IU)	1000	Vitamin B_12_ (µg)	80.0
Methionine (g)	4.1	Arginine (g)	13.0
Vitamin E (mg)	90.0	Pantothenate (mg)	30.0
Tryptophan (g)	3.0	Phenylalanine (g)	10.0
Vitamin K_3_ (mg)	3.0	Folic acid (mg)	5.0
Threonine (g)	7.4	Tyrosine (g)	7.8
Vitamin B_1_ (mg)	21.0	Nicotinic acid (mg)	133.0
Isoleucine (g)	17.5	Choline (mg)	2750.0
Vitamin B_2_ (mg)	16.0	Biotin (mg)	0.4
Valine (g)	11.0		
Calcium (g)	10.0	Potassium (g)	9.4
Iron (mg)	250.0	Cobalt (mg)	2.0
Phosphorus total (g)	8.17	Sodium (g)	2.2
Manganese (mg)	100.0	Iodine (mg)	1.0
Phosphorus saturated (g)	4.5	Chlorine (g)	2.5
Zinc (mg)	76.9	Selenium (mg)	0.5
Magnesium (g)	3.0	Sulfur (g)	1.9
Copper (mg)	21.3		

**Table 2:** Characteristic of experimental groups.

	CON	ZnM	ZnN	*p* Value *
Mass start [g]	134.2 ± 13.8 ^a^	121.9 ± 8.6 ^a,b^	114.5 ± 6.4 ^b^	0.0028
Mass end [g]	231.0 ± 13.8	230.1 ± 17.2	230.4 ± 10.2	n.s.
Mass increase [g]	96.7 ± 10.3 ^a^	108.3 ± 12.5 ^a,b^	115.9 ± 9.7 ^b^	0.0075
Liver [g]	6.8 ± 0.8	6.2 ± 0.3	6.2 ± 0.5	n.s.
Kidneys [g]	1.6 ± 0.2	1.7 ± 0.1	1.8 ± 0.1	n.s.
Spleen [g]	0.6 ± 0.1	0.6 ± 0.2	0.6 ± 0.1	n.s.
Heart [g]	1.1 ± 0.1	1.1 ± 0.1	1.1 ± 0.1	n.s.

* statistically significant difference (α = 0.05). ^a,b^—homogenous groups in rows (α = 0.05), n.s.—not significant, CON—control group; ZnM—group receiving zinc in microparticles; ZnN—group receiving zinc in nanoparticles.

**Table 3:** The efficiency of tumor induction.

Group	Number of Tumors per Individual—Range	Tumor Incidence of Necropsy [%]	Tumor Weight Ranges [g]	Tumor Weight Mean [g]
CON	2–9	100	0.01–7.80	0.89 ± 0.52 ^a^
ZnM	1–6	100	0.06–7.41	0.68 ± 0.66 ^a,b^
ZnN	0–3	88	0.01–1.79	0.40 ± 0.34 ^b^

^a,b^—homogenous groups in rows (α = 0.05); CON—control group; ZnM—group receiving zinc in microparticles; ZnN—group receiving zinc in nanoparticles.

**Table 4:** Fatty acids (FA) profile in serum.

Fatty Acid [μg/mL]	CON	ZnN	ZnM	*p* Value *
SFA				
C10:0	10.19 ± 8.63	8.14 ± 5.51	4.04 ± 1.70	n.s.
C12:0	12.57 ± 8.52	10.17 ± 3.8	7.00 ± 1.74	n.s.
C14:0	24.64 ± 25.68	18.64 ± 6.36	15.48 ±3.53	n.s.
C15:0	13.58 ± 4.11 ^a^	10.12 ± 2.97 ^a,b^	9.15 ± 1.60 ^b^	0.022
C16:0	616.7 ± 285.6	543.1 ± 118.9	677.3 ± 145.6	n.s.
C17:0	16.55 ± 3.55 ^a^	13.04 ± 1.88 ^b^	16.01 ± 1.86 ^a,b^	0.026
C18:0	359.1 ± 66.9 ^a^	393.1 ± 85.4 ^a^	510.22 ± 76.5 ^b^	0.002
∑ SFA	1123 ± 393	1051 ± 134	1271 ± 110	n.s.
MUFA				
C16:1 n-9	9.83 ± 3.10	9.18 ± 3.64	7.11 ± 1.18	n.s.
C16:1 n-7	60.33 ± 13.61 ^a^	45.63 ± 30.97 ^a,b^	25.60 ± 3.89 ^b^	0.007
C18:1 n-9 OL	229.5 ± 116.6	196.5 ± 50.5	243.5 ± 32.5	n.s.
C18:1 n-7	33.90 ± 10.94	32.29 ± 5.74	40.77 ± 5.25 ^a^	n.s.
∑ MUFA	333.6 ± 140.0	283.6 ± 86.5	317.0 ± 37.4	n.s.
PUFA				
C18:2 n-6 LA	588.3 ± 20.5	578.0 ± 154.5	632.4 ± 72.5	n.s.
C18:3 n-6 GLA	18.55 ± 5.07 ^a^	18.62 ± 9.33 ^a^	10.18 ± 2.33 ^b^	0.019
C18:3 n-3 ALA	46.69 ± 15.30 ^a^	36.92 ± 17.98 ^a,b^	27.13 ± 3.04 ^b^	0.032
C20:3 n-6 DGLA	6.30 ± 3.28	5.72 ± 1.07	7.53 ± 1.65	n.s.
C20:4 n-6 AA	878.7 ± 166.9	859.8 ± 156.0	820.9 ± 125.7	n.s.
C20:5 n-3 EPA	54.67 ± 24.89 ^a^	32.59 ± 12.05 ^b^	20.05 ± 6.02 ^b^	0.001
C22:5 n-6 DPA	16.06 ± 7.72	15.13 ± 5.58	14.15 ± 4.71	n.s.
C22:6 n-3 DHA	170.2 ± 44.6	139.5 ± 34.3	138.1 ± 27.7	n.s.
∑ PUFA	1779 ± 416	1686 ± 336	1670 ± 185	n.s.
n-3	271.6 ± 68.2 ^a^	209.0 ± 59.1 ^a,b^	185.2 ± 27.48 ^b^	0.013
n-6	1507 ± 360	1477 ± 288	1485 ± 137	n.s.

Data are presented as mean values ± standard deviation. * statistically significant difference (α = 0.05), ^a,b^—homogenous groups in rows (α = 0.05), n.s.—not significant; CON—control group; ZnM—group receiving zinc in microparticles; ZnN—group receiving zinc in nanoparticles; MUFA—monounsaturated fatty acids; PUFA—polyunsaturated fatty acids; SFA—saturated fatty acids; Σ—sum of SFA, MUFA or PUFA, respectively.

**Table 5:** FA profile [%] in experimental groups.

	Control	ZnN	ZnM	*p* Value *
SFA [%]	32.87 ± 2.06 ^b^	34.00 ± 3.50 ^b^	38.41 ± 1.87 ^a^	<0.001
MUFA [%]	10.32 ± 1.31	9.41 ± 1.53	9.83 ± 0.91	n.s.
PUFA [%]	56.81 ± 3.14 ^a^	56.60 ± 2.90 ^a^	51.76 ± 2.00	0.001
n-6/n-3 PUFA	5.64 ± 0.90 ^b^	7.34 ± 1.21 ^a^	8.16 ± 1.28 ^a^	<0.001
(MUFA + PUFA)/SFA	1.92 ± 0.15 ^a^	1.87 ± 0.24 ^a^	1.57 ± 0.12 ^b^	0.002
PUFA/SFA	1.62 ± 0.16 ^a^	1.60 ± 0.21 ^a^	1.32 ± 0.11 ^b^	0.002
PI	194.2 ± 16.5 ^a^	186.9 ± 13.1 ^a^	165.0 ± 11.2 ^b^	0.001

Data are presented as mean values ± standard deviation. * statistically significant difference (α = 0.05), ^a,b^—homogenous groups in rows (α = 0.05), n.s.—not significant; CON—control group; ZnM—group receiving zinc in microparticles; ZnN—group receiving zinc in nanoparticles; MUFA—monounsaturated fatty acids; PUFA—polyunsaturated fatty acids; SFA—saturated fatty acids; PI—peroxidability index.

**Table 6:** Content of cholesterol, squalene and oxysterols in serum of rats [μg/mL].

[μg/mL]	CON	ZnN	ZnM	*p* Value *
Squalene	19.79 ± 12.39	11.78 ± 3.02	18.95 ± 16.80	n.s.
Cholesterol	2006 ± 1091 ^a^	979 ± 115 ^b^	1573 ± 525 ^a,b^	0.027
7K-Ch	5.12 ± 2.60 ^a^	2.64 ± 0.79 ^b^	3.70 ± 1.64 ^a,b^	0.042
7α-OH-Ch	1.81 ± 1.00 ^a^	0.77 ± 0.17 ^b^	1.18 ± 0.43 ^a,b^	0.013
7β-OH-Ch	4.69 ± 2.25 ^a^	2.04 ± 0.32 ^b^	2.87 ± 1.00 ^b^	0.004
5,6βE-Ch	5.84 ± 3.03 ^a^	2.70 ± 0.63 ^b^	4.20 ± 1.44 ^a,b^	0.016
∑ COPs	17.45 ± 8.53 ^a^	8.15 ± 1.51 ^b^	11.95 ± 4.37 ^a,b^	0.011
COPs/Ch [%]	0.94 ± 0.33	0.83 ± 0.10	0.75 ± 0.06	n.s.

Data are presented as mean values ± standard deviation. * statistically significant difference (α = 0.05), ^a,b^—homogenous groups in rows (α = 0.05), n.s.—not significant; CON—control group; ZnM—group receiving zinc in microparticles; ZnN—group receiving zinc in nanoparticles; 7-ketocholesterol (7K-Ch); 7α-hydroxycholesterol (7α-OH-Ch); 7β-hydroxycholesterol (7β-OH-Ch); 5β,6β-epoxycholesterol (5,6βE-Ch), COPs—cholesterol oxidation products; ∑ COPs—sum of COPs.
